# The association between household biomass fuel use and leukocyte telomere length among toddlers in Bhaktapur, Nepal

**DOI:** 10.1038/s41370-022-00474-1

**Published:** 2022-09-22

**Authors:** Ram K. Chandyo, Catherine Schwinger, Ingrid Kvestad, Manjeswori Ulak, Suman Ranjitkar, Merina Shrestha, Linda Vy Nguyen, Diana Corona-Perez, Immaculata DeVivo, Laxman Shrestha, Tor A. Strand

**Affiliations:** 1 Department of Community Medicine, Kathmandu Medical College, Kathmandu, Nepal; 2 Centre for Intervention Science in Maternal and Child Health, Centre for International Health, Department of Global Public Health and Primary Care, University of Bergen, Bergen, Norway; 3 Regional Centre for Child and Youth Mental Health and Child Welfare, NORCE Norwegian Research Centre, Bergen, Norway; 4 Department of Research, Innlandet Hospital Trust, Lillehammer, Norway; 5 Department of Child Health, Institute of Medicine, Tribhuvan University, Kathmandu, Nepal; 6 Department of Epidemiology, Harvard T.H. Chan School of Public Health, Boston, MA USA; 7 Channing Division of Network Medicine, Brigham and Women’s Hospital, Boston, MA USA

**Keywords:** Biomass fuel, Leukocyte telomere length, Children, Nepal

## Abstract

**Background:**

Biomass fuels are still in use for cooking by many households in resource poor countries such as Nepal and is a major source of household air pollution (HAP). Chronic exposure to HAP has been shown to be associated with shorter telomere length in adults.

**Objectives:**

To measure the association between exposure related to household biomass fuel in infancy and leukocyte telomere length (LTL) at 18–23 months of age among 497 children from Bhaktapur, Nepal.

**Methods:**

In a prospective cohort study design, we have collected information on household cooking fuel use and several clinical, anthropometric, demographic, and socioeconomic variables. We estimated the association between biomass fuel use and the relative LTL in multiple linear regression models.

**Results:**

Most of the families (78%) reported liquified petroleum gas (LPG) as the primary cooking fuel, and 18.7% used biomass. The mean relative (SD) LTL was 1.03 (0.19). Children living in households using biomass fuel had on average 0.09 (95% CI: 0.05 to 0.13) units shorter LTL than children in households with no biomass fuel use. The observed association was unaltered after adjusting for relevant confounders. The association between LTL and biomass use was strongest among children from households with ≤2 rooms and without separate kitchen.

**Significance:**

Exposure to biomass fuel use in early life might have consequences for longevity, and risk of chronic illnesses reflected in shortening of the telomeres. Our findings support the ongoing effort to reduce exposure to biomass fuel in low-resource settings.

**Impact statements:**

Biomass for cooking is a leading source of household air pollution in low and middle-income countries, contributing to many chronic diseases and premature deaths. Chronic exposure to biomass fuel through oxidative stress and inflammation has been associated with a shortening of the telomeres, a “biological marker” of longevity. This prospective cohort study describes the association between household biomass fuel use and leukocyte telomere length among 497 toddlers. Leukocyte telomere length was significantly shorter among children living in households with biomass fuel than in children from homes where mainly LPG was used for cooking.

**Clinical Trial registration:**

Clinicaltrials.gov: NCT02272842, registered October 21, 2014, Universal Trial Number: U1111-1161-5187 (September 8, 2014)

## Introduction

Biomass cooking fuel, mainly used in the form of firewood, coal, saw dust, animal dung or crop residues, is the main source of household air pollution (HAP) in many low- and middle-income countries (LMIC) [1]. The burning of biomass results in toxic pollutants such as particulate matter (PM), methane, black carbon, nitrous oxide, and carbon monoxide [2]. Globally more than 40% of households in LMICs are still dependent on biomass for cooking primarily due to poverty and the lack of alternative cleaner fuels [3].

Chronic exposure to HAP has been linked to several morbidities such as respiratory diseases (pneumonia, COPD, lung cancer, pulmonary tuberculosis), cardiovascular diseases (ischemic heart disease, stroke, hypertension), as well as cataracts [4, 5]. HAP was estimated to be responsible for 91.5 million DALY leading to 2.3 million deaths in 2019, which is 4% of all deaths [3]. Women and children are particularly vulnerable to HAP as they spend more time cooking indoors being directly exposed [6]. HAP during pregnancy has been associated with adverse pregnancy outcomes such as miscarriage, eclampsia, prematurity, and low birth weight [4], whereas exposure during early childhood has been associated with pneumonia [7], poor growth and impaired cognitive development [8, 9].

In Nepal, HAP, particularly from biomass fuel use, was listed as the second-largest contributor to the total disease burden [10]. This burden, however, varied substantially according to geographical locations and socio-economic status [11]. In 2015, the Government of Nepal initiated campaigns for cleaner energy for cooking and smokeless stoves to reduce the use of household biomass fuel from 75% to 30% within 2030 (SDG goal 7, target 7.1.2) [12].

Although the exact mechanisms on how HAP leads to chronic disease and premature death are unclear, recent evidence suggests that a possible pathway could be through shortening of the leukocyte telomere length (LTL) [13]. Telomeres are crucial to maintain chromosome stability and consist of repetitive DNA and protein complexes [14]. Telomere length serves as a “biological clock” and the shorter length is considered a propensity for an early onset of non-communicable diseases such as diabetes [15], obesity [16], COPD [17], and cancer [18]. LTL is naturally shortened with each cellular replication but may also be prematurely shortened due to various diseases [19] and environmental factors including smoking [20]. The process of telomere shortening may accelerate due to oxidative stress and inflammation [21], and both conditions are common during chronic exposure to HAP [22].

Two available systematic reviews and meta-analyses based on observational studies among adults confirmed a negative association between air pollution and telomere length [23, 24]. For HAP, a study among rural Chinese women showed that those using biomass fuel for cooking with a high exposure to PM_2.5_ and black carbon had shorter LTL than women with lower exposure [25]. Although LTL at birth has been shown to be associated with exposure to toxic pollutants during pregnancy [26], it remains unclear if the observed associations between biomass fuel use and telomere length can also be seen at young ages. In our study, we estimate the association between biomass use as a primary kitchen fuel assessed during infancy and LTL measured at 18–23 months of age in children from Bhaktapur, Nepal.

## Methods

### Study design and population

The study was conducted in Bhaktapur municipality which is predominantly a residence of the Newar ethnic group. The area also has an immigrant population from other parts of the country, many of whom work at carpet factories. Bhaktapur is one of four municipalities in the Bhaktapur district and has a population of approximately 79,000 with an annual growth rate of 3.3%. The municipality typically has clusters of old brick houses and is one of the most densely populated cities in Nepal (11,430 inhabitants/km^2^) [27]. Agriculture, small family businesses, services at the government or private organizations, and daily wage jobs are the main sources of income [28].

There are many brick kilns located at the outskirts of Bhaktapur municipality, which are functioning mainly during the winter and spring season (from October to May) and contribute to the poor ambient air quality due to the use of coal and firewood for burning bricks [29]. Additionally, relatively high CO_2_ emissions from vehicles are also common in Bhaktapur with an estimated emission load of 3310 tones/year [30]. Communal firing spots (ash kilns) are still in use in close proximity to residential houses in some areas of Bhaktapur where ceramic raw pots are stacked in layers of hay and then covered by ash for approximately 4 days to maintain the required temperature. All these factors may contribute to the high ambient air pollution in Bhaktapur, reflected in recordings of a mean 24 h PM_2.5_ concentration exceeding 15 μg/m^3^, which is the cut-off in the air quality index by the World Health Organization (WHO) [31].

### Enrollment procedure and definitions

For the current analyses, we used data of children enrolled in a community-based, randomized, double-blind, placebo-controlled trial assessing the effect of daily vitamin B12 supplementation over a period of 12 months on neurodevelopment and growth [32]. Between April 2015 and February 2018, 600 children aged 6–11 months, mildly stunted (length-for-age Z-score of <−1) and available written consent from one of the parents were enrolled into the study and weekly followed up for one year. We targeted infants with length-for-age Z-score of <−1 SD because they constitute a high risk population [33] with regard to poor cognitive development, growth failure, and malnutrition. Screening of children for the eligibility to enroll in the study were done at different vaccination clinics and through a household survey. Infants who were eligible, were requested to visit at the study clinic at 6 months of age for re-assessment. Children were not enrolled if they had one of the following conditions: 1. Severe systemic illnesses requiring hospitalization, 2. Ongoing or planned multivitamin supplement intake that included vitamin B_12_, 3. Severe anemia (Hb < 7 g/dl), 4. Ongoing acute infection such as fever, cough or diarrhea that requires medical treatment, and 5. Severe malnutrition (weight-for-length Z-score < −3). The main results of the project have been reported elsewhere. In short, the intervention resulted in a substantially improved vitamin B12 status but did not improve growth or neurodevelopment neither at 18–23 months [34] nor 30–47 months of age [35].

Regular anthropometric measurements of children were carried out by trained field workers using digital weighing scales and infantometers (Seca, Germany). All measurements were taken twice, and the mean of these measurements were used in the analyses. For parental measurements, we used a stadiometer for height (Prestige, Hardik Medi Tech, India) and a portable electronic scale for weight (Salter, UK).

Baseline information covering socio-demographic, education and family characteristics were obtained from the caretakers at enrollment. We used a modified WAMI index to indicate socio-economic status, which stand for Water and sanitation, availability of Assets at the household and Maternal education as described by others [36]. The original WAMI index also includes household income, since we did not have information on income, we only included the remaining 3 indicators. The WAMI index ranges from 0–1 where a higher score indicates a better socio-economic condition.

We also collected information to describe the quality of indoor air in the households during enrollment. This included availability of a separate kitchen, smoking habits inside the house, and primary kitchen fuel use. Fuel use was defined as biomass if the family reported a current primary use of one of the following fuels for cooking: firewood, saw dust, cow dung, straw, or any crop residues.

### Blood collection

One year after enrollment, when the children were 18–23 months of age, we collected ~3 ml blood into vials containing EDTA as anticoagulant (Eppendorf, Germany). The hemoglobin concentration was measured using HemoCue immediately after blood collection (HemoCue, Vedbæk, Denmark). Anemia at 6–11 months (enrollment) and 18–23 months (one year follow up period) of age was defined when hemoglobin concentration was <11 g/dl as per the WHO guidelines. The sample was centrifuged at 2000 to 2500 at room temperature for 10 minutes and after separation from the plasma stored at −70 degree Celsius until analyses during November 2020.

### Leukocyte telomere length assay

The LTL analysis was done in the Research Laboratory at Harvard School of Public Health, Boston, USA (De Vivo lab). Five nanograms of genomic DNA were dried down in each well of a 384-well plate and resuspended in 10 µL of either the telomere or 36B4 PCR reaction mixture and stored in 4 ^°^C up to 6 hours. The telomere reaction mixture consisted of 1x Thermo Fisher PowerUP SYBR Master Mix, 2.0 mM of DTT, 270 nM of Tel-1b primer, and 900 nM of Tel-2b primer. The reaction proceeded for 1 cycle hold at 50 °C for 2 minutes and at 95 °C for 2 minutes, followed by 35 cycles at 95 °C for 15 seconds, and 54 °C for 2 minutes. The 36B4 reaction consisted of 1x Thermo Fisher PowerUP SYBR Master Mix, 300 nM of 36B4U primer, and 500 nM of 36B4D primer. The 36B4 reaction proceeded for 1 cycle hold at 50 °C for 2 minutes and at 95 °C for 2 minutes, followed by 40 cycles at 95 °C for 15 seconds, and 58 °C for 1 minute and 10 seconds. All samples for both the telomere and single-copy gene (36B4) reactions were performed in triplicate on different plates. Each 384-well plate also contained a 6-point standard curve from 0.625 ng to 20 ng using pooled buffy-coat derived genomic DNA.

The standard curve assessed and compensated for inter-plate variations in PCR efficiency. The slopes of the standard curve for both the telomere and 36B4 reactions were −3.33 ± 0.33 and the linear correlation coefficient (*R*^2^) values for both reactions were over 0.99. The T/S ratio (-dCt) for each sample was calculated by subtracting the average 36B4 Ct value from the average telomere Ct value. The relative T/S ratio (-ddCt) was determined by subtracting the T/S ratio value of the 5 ng standard curve point from the T/S ratio of each unknown sample [37]. Quality control samples were interspersed throughout the test samples in order to assess inter-plate and intra-plate variability of threshold cycle (Ct) values. A combined inter- and intra-assay coefficient of variation (CV) calculated from the relative T/S ratio (-ddCt) of quality control samples is 8.1%. The LTL are expressed in relative units with a mean of approximately 1.

### Statistical analysis

All collected data were checked manually by the study supervisors and double-entered in either the Microsoft Access database or a cloud based mobile data collection platform Iformbuilder (https://www.zerionsoftware.com/iformbuilder). Data are presented as numbers and percentages (%) for categorical variables or means and standard deviations (SD) for continuous variables. We produced density plots of LTL separately for households reporting biomass fuel use for cooking and for those who did not, using the Epanechnikov Kernel function. Multiple linear regression analyses adjusting for potential confounding variables were done to estimate the association between LTL and biomass fuel use. Household and child characteristics (gender, stunting, exclusive breastfeeding, low birth weight, age of child and mother, presence of indoor smoking, anemia and WAMI index) were selected as potential confounders and included in the regression model based on clinical and epidemiological relevance as well as the availability of data. We also re-ran the regression analyses using the same covariates as in the main regression model in the following pre-defined subgroups: birthweight (<2500 g vs. ≥2500 g), exclusive breastfeeding (<3 months vs. ≥3 months), stunting (HAZ < −2 SD vs. ≥−2 SD), underweight (WAZ < −2 SD vs. ≥−2 SD), number of rooms in the house (≤2 rooms vs. >2 rooms), separate kitchen and bedroom and practice of indoor smoking (yes vs. no). We assessed whether these variables significantly modified the association between biomass and LTL by including interaction terms in the regression models. Regression coefficients and 95% CIs from these subgroup analyses were displayed in a forest plot. In the same manner, we present conditional regression coefficients from quantile regression analyses for the following percentiles of LTL: 95, 90, 80, 70, 60, 50, 40, 30, 20, 10, and 5. We used these quantile regression models to assess if there are any differences in the associations of biomass cooking fuel use and LTL in the different percentiles of the LTL distribution. All data analyses were done using Stata 16 (StataCorp LLC).

### Ethics

We obtained ethical clearance from the Nepal Health Research Council (NHRC #233/2014), and from the Regional Committee for Medical and Health Research Ethics (REC # 2014/1528), Norway. After informing the caretaker (usually the mother) about the details of the study, we received written consent. During the study period, children with acute illnesses were treated according to the CB-IMNCI guidelines.

## Results

LTL was measured in the last 497 (87%) of the 574 children who completed the one-year follow-up of the main trial. Almost half of the children were female, 6–7 months of age at enrollment, or living in joint families (Table 1). There were no substantial differences in baseline characteristics between children for whom we did LTL analysis (*n* = 497) and those we did not (*n* = 77). Upon enrolment, all but 14 mothers were still breastfeeding, and 52% had initiated breastfeeding within 1 hour of delivery. One in every 4 children had been introduced to solid or semi-solid foods by 2 months of age. Although the prevalence of anemia was high when the children were 6–11 months (64%), it decreased to 26% when children reached 18–23 months of age. Among the anemic children, both at baseline and at the one year follow up, 60% were mildly anemic (hemoglobin concentration 10–10.9 g/dL) and 40% had moderate anemia (hemoglobin concentration 7–9.9 g/dL).

**Table 1:** Baseline and follow up information of 497 Nepalese infants in Bhaktapur, Nepal.

Baseline characteristics	Baseline (6–11 m) *n* (%)^a^	
	Biomass 93 (18.7)	No biomass 404 (81.3)
Infant characteristics		
Mean age of child (months), mean ± SD	7.8 ± 1.8	7.9 (1.7)
Female child	42 (45)	199 (49)
First born (birth order)	43 (46)	194 (48)
Low birth weight (<2500 g)^b^	19 (20)	74 (18)
Preterm delivery (≤37 weeks of gestation)	11 (11.8)	42 (10.4)
Delivery by cesarean section	32 (34.4)	133 (32.9)
No breastfeeding at the time of enrollment	2 (2.1)	12 (2.9)
Exclusive breastfeeding for ≥3 months	40 (43)	194 (48)
Underweight (weight for age z-score ≤ −2)	19 (20.4)	69 (17.1)
Stunting (length for age z-score ≤ −2)	38 (40.8)	117 (28.9)
Wasting (weight for length z-score ≤ −2)	4 (4.3)	12 (2.9)
Anemia (hemoglobin <11 g/dl)	66 (70.9)	252 (62.4)
Family and household features		
Mother’s age, mean ±	28.2 ± 4.5	27.4 (4.6)
Mothers who completed secondary school or above	55 (59.1)	260 (64.4)
Mothers who work (daily wage, business, or service)	29 (31.2)	177 (43.8)
Underweight mothers (<18.5 kg/m^2^ BMI)	1 (1.1)	25 (6.2)
Family staying in joint family	71 (76.3)	176 (43.6)
Family residing in rented house	22 (23.6)	210 (51.9)
Kitchen and bedroom in same room	28 (30.1)	210 (51.9)
Family with indoor tobacco smoking practice	33 (35.5)	214 (52.9)
Family having own land	63 (67.7)	179 (44.3)
WAMI index^c^	0.64 0.01)	0.61 (0.14)
Receiving remittance from abroad	4 (4.3)	44 (10.8)

^a^Values are *n* (%) unless otherwise specified.

^b^Among 480 newborns where birth weights were recorded.

^c^Water and sanitation, availability of Assets at household and Maternal education and household Income.

**Fig. 1:**
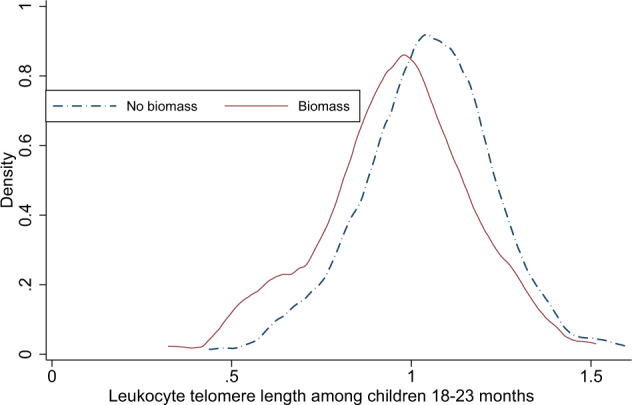
Distribution of leukocyte telomere length among 18–23 months old children  in Bhaktapur, Nepal by biomass fuel use in the household. The group from households using biomass fuel is indicated by solid lines, the group from houshoulds using other cooking fuels is indicated by dotted lines.

Most of the families (78%) reported use of LPG as the primary fuel for cooking, and 18.7% families used biomass fuel. One in every second families reported practice of indoor smoking, probably due to high numbers of fathers who smoke regularly (35%). The LTL was normally distributed, and the relative mean (SD) was 1.03 (0.19) ranging from 0.32 to 1.59. The distribution of LTL of the children is graphically presented in Fig. 1 separately for those from biomass-use households and other households. In the unadjusted regression analysis, children from biomass fuel use households had 0.08 units shorter LTL (95% CI: 0.04 to 0.12) than children from households that did not use biomass fuel (Table 2). This association was unaltered after including potential confounders in the regression model.

**Table 2:** Regression analysis between leukocyte telomere lengths and different household and family characteristics among 497 children in Bhaktapur, Nepal.

Variable	Crude coefficient (95% CI)	Adjusted coefficient (95% CI)^a^
Biomass fuel use (ref. no)	−0.08 (−0.12, −0.04)	−0.09 (−0.13, −0.04)
Low birth weight (ref. no)	0.02 (−0.01, 0.07)	0.02 (−0.03, 0.06)
Age of children (in months)	0.00 (−0.01, 0.01)	0.08 (−0.05, 0.23)
Age of mother (in years)	−0.00 (−0.01, 0.03)	−0.09 (−0.22, 0.06)
Exclusive breastfeeding for 3 months of more (ref no)	−0.02 (−0.05, 0.01)	−0.02 (−0.05, 0.01)
WAMI index^b^	−0.03 (−0.14, 0.09)	−0.01 (−0.14, 0.11)
Gender (ref. male)	0.05 (0.01, 0.08)	0.04 (0.01, 0.08)
Stunting (<−2 HAZ) (ref. no)^c^	0.01 (−0.05, 0.02)	0.00 (−0.04, 0.04)
Anemia (Hb < 11 g/dL) (ref. no)^c^	0.03 (−0.00, 0.07)	0.04 (0.01, 0.08)
Indoor smoking (ref. no)	0.01 (−0.02, 0.04)	0.00 (−0.03, 0.04)

^a^Adjusted for variables listed in the table, mother occupation, household ownership of land, availability of separate kitchen, family staying on rent and joint family.

^b^Water and sanitation, availability of Assets at household and Maternal education and household Income).

^c^At 6–11 months of age.

**Fig. 2:**
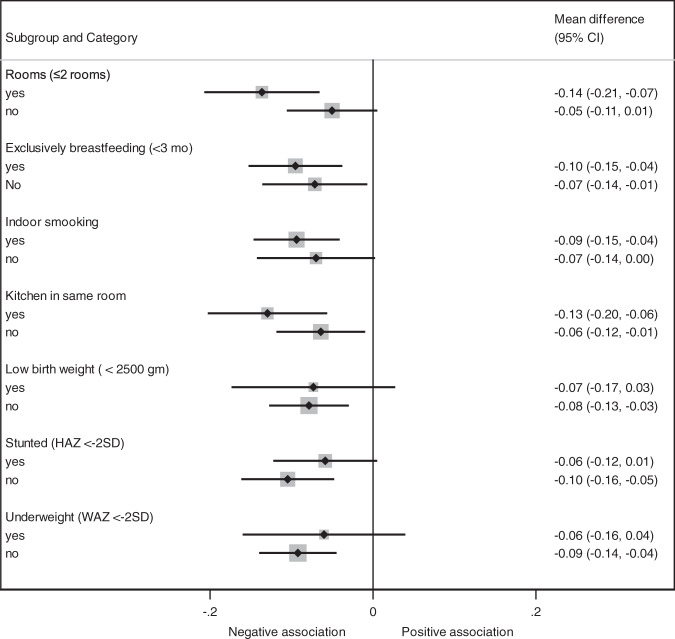
Association between biomass cooking fuel and leukocyte telomere length in pre-defined subgroups. Regression coefficients (mean difference) and 95% confidence intervals (95%CI) are from linear regression models, adjusted for the WAMI index, age, sex of the child, maternal age and indoor smoking.

**Fig. 3:**
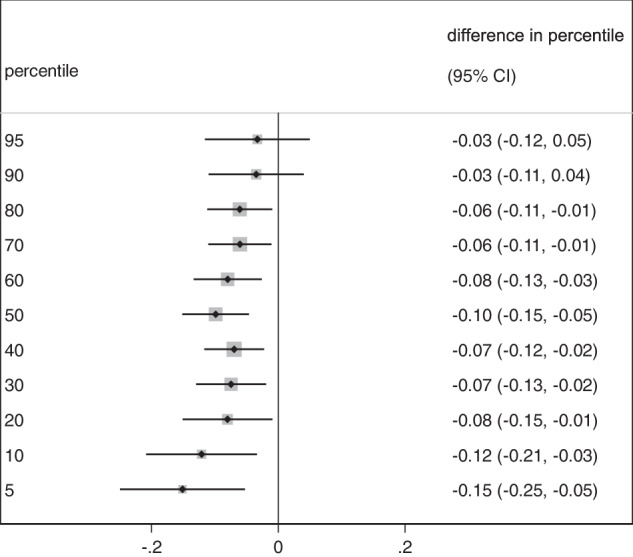
Association between biomass cooking fuel and pre-defined percentiles distribution of leukocyte telomere length. Regression coefficients (ES) and 95% confidence intervals are from quantile regression models adjusted for WAMI, age, and sex of the child, maternal age, and indoor smoking.

We also depicted the associations between biomass fuel use and LTL in several pre-defined subgroups (Fig. 2). Nutritional status indicators of the children such as exclusive breastfeeding for 3 months, underweight, stunting and birth weight did not modify the association. The corresponding p-values for the interaction were 0.5, 0.41, 0.23, 0.9, respectively. However, the association between biomass use and LTL was stronger in children from households with ≤2 rooms in the house (*p*-value for interaction 0.07), or who did not have separate kitchen (*p*-value for interaction 0.16). In the quantile regression analyses, the association between biomass fuel use and LTL showed a tendency of an increase in percentile difference in LTL towards the lower end of the LTL distribution. In other words, there was a stronger association between biomass fuel use and LTL in those with a shorter LTL compared to those with a longer LTL (Fig. 3).

## Discussion

In the present prospective cohort of Nepalese toddlers, approximately one in five households used biomass as primary fuel for cooking. We found that the LTL was shorter among children from these households compared to in children living in households using primarily LPG as cooking fuel. The negative impact of HAP mainly driven by biomass fuel use, is well established for several adverse health outcomes in both children and adults [3]. This is, to the best of our knowledge, the first report describing an association between biomass fuel use and LTL in young children.

The available literature on the relationship between telomere length and exposure to air pollutants is primarily from studies measuring the ambient air quality at a population level [24, 38]. In our study, we have individual data on the exposure to biomass fuel from each household and LTL measured when the children were 18–23 months. We also have comprehensive data on other exposures such as growth, household, and socioeconomic characteristics. The longitudinal design, the ability to measure several potential confounding variables, and the relatively large sample size, provide strengths to our findings of an association between the exposure of biomass fuel and LTL in young children [3, 39, 40].

An interesting observation in our analyses is that there were few other factors associated with LTL. In the adjusted model, we found that girls had on average longer LTL than boys, and that those with anemia at enrolment had longer LTL one year later than those who did not have anemia. Age was not associated with LTL, which is probably due to the narrow age range (18 to 23 months) when the blood sample was taken. Our finding of longer telomere lengths in female than male children is expected and was also found in a cohort study in 4000 children in New Zealand where the telomere length was measured in 4-year-olds [41]. Similar gender wise variation in LTL and an association with lower life expectancy at the age of 40 years was described among UK Biobank participants [42].

In the subgroup analyses, the negative association between biomass fuel use and LTL was even stronger in children living in households with fewer than 3 rooms as well as in those who did not have separate kitchen and bedroom. Both not having a separate kitchen and having few rooms in the household is likely to contribute to increased HAP exposure which could partly explain the modified association [43]. The quantile regression analysis (Fig. 3) indicates a stronger association between LTL and HAP in the lower than higher LTL distributions which is an indication of exposure to HAP mostly affected children with lower LTL percentiles.

The use of biomass fuels for cooking is still very common in LMIC including Nepal, particularly in rural areas [3]. Our study site, although located in an urban and peri-urban area, is an agricultural community, and biomass fuels, mainly crop residues, were still used by 18.7% of all households. However, the use of biomass fuel seems to be decreasing in Bhaktapur; while 25% of households reported to use biomass fuel for cooking in a survey in 2006–2007 [39], there were only 5% of the households reporting the same in a survey in 2018–2020 [40]. At the beginning of the study, our site was severely affected by a large earthquake. During the re-construction period, many people lived in temporary shelters with limited access to basic needs. Moreover, blockades at the Indian border for more than 6 months led to restricted LPG availability. Both the consequences from the earthquake and blockades could be causes for an increased biomass fuel use at the study site.

Although we collected data after the child was born, it is likely that these children were exposed to toxic pollutants from biomass fuel use already during the intra-uterine period. Both exposure on HAP questionnaire and LTL measurements were done in one time point, our study can not distinguish timing of exposure whether it was during pregnancy or after the birth. Indeed, exposure to such pollutants during pregnancy has also shown to be associated with shorter telomere lengths at birth [26] and with subsequent higher systolic blood pressure at four years of age [44]. Beside genetic and environmental determinants, variations in telomere length could also be due to exposure to oxidative stress and chronic inflammation in utero as a counter regulatory effect of the telomerase which is a reverse transcriptase enzyme maintaining telomere length [45].

This is a secondary analysis from a nutritional randomized trial enrolling mildly stunted infants [34] and caution is required for the generalizability of our findings. Micronutrient status may also affect LTL [46]; however, we did not find any association between vitamin B12 supplementation and the LTL. We used biomass fuel use for cooking as a proxy for HAP and lack information on the specific exposure of pollutants such as PM_2.5_, black carbon, or nitrous oxide which may be regarded as a limitation to our study. We also lack information on secondary cooking fuels in the households, as well as fuel used for heating. We know however, that in this area such energy sources are primarily used for cooking purpose [47] limiting the exposure to secondary fuel use for heating. Still the lack of information on the secondary cooking fuels may lead to exposure misclassification which we believe may bias the results towards the null [48]. Moreover, despite adjusting for several relevant confounding variables, there is a possibility that the observed association is due to residual confounding. Since we asked about primary fuel use at one time point (when children were 6–11 months of age), there could have been changes in stove/fuel use during the course of the study.

In this community-based cohort, children who were living in households with biomass fuel use had substantially shorter LTL compared with children from households where mostly LPG were used as the primary kitchen fuel. The immediate and long-term possible consequences of these shorter LTL should be explored in future studies.

## Supplementary information

Reporting Checklist

## Data Availability

Data available on request. To meet ethical requirements for the use of confidential patient data, requests must be approved by the Nepal Health Research Council (NHRC) and the Regional Committee for Medical and Health Research Ethics in Norway. Requests for data should be sent to the authors, by contacting, the Department of Global Health and Primary Care at the University of Bergen (post@igs.uib.no) or Child Health Research Project, Department of Child Health, IOM, Nepal (chrp2015@gmail.com).
